# Effects of communication, trust, and respect on shared decision-making: insights from healthcare providers in Chinese public hospitals

**DOI:** 10.3389/fmed.2025.1577276

**Published:** 2025-06-18

**Authors:** Lu Chen, Huijun Chu, Hui Wang, Liang Du, Yumeng Gao, Jie Shen

**Affiliations:** ^1^Medical Records and Statistics Department, Shanghai Sixth People’s Hospital, Shanghai Jiao Tong University School of Medicine, Shanghai, China; ^2^Department of Medical, Putuo People’s Hospital, School of Medicine, Tongji University, Shanghai, China; ^3^School of Public Health, Fudan University, Shanghai, China; ^4^Shanghai Public Health Clinical Center, Fudan University, Shanghai, China

**Keywords:** shared decision-making, communication, trust, respect, public hospitals, China

## Abstract

**Background:**

Shared decision making (SDM) could significantly enhance health knowledge, treatment adherence, and doctor-patient relationship, but multifaceted barriers have influenced the implementation of SDM worldwide. There are now few studies on SDM process from the perspective of healthcare workers who often act as the initiators of SDM. Focusing on healthcare providers, this study aimed to explore the mechanism by which provider-patient communication, trust, and respect influenced SDM within the context of China’s three-tier public hospital system.

**Methods:**

A stratified sampling was employed to survey doctors and nurses from public hospitals in Shanghai, China. The questionnaire included respect, patient-provider communication, trust, SDM, and socio-demographic information. Structural equation modeling was used to examine the study hypotheses, after controlling demographics covariates.

**Results:**

There were 778 participants included in this study. The constructs in our study exhibited good reliability and validity, and the SEM demonstrated good fit (CFI = 0.978, TLI = 0.974, RMSEA = 0. 039, SRMR = 0.036). Provider-patient communication and trust were significant factors influencing SDM (*p* < 0.001), and R-square for regression models were all more than 30%. Additionally, trust between providers and patients mediated the relationship between communication and SDM (effect = 0.221, 95% CI: 0.133–0.359), and the mediating effect accounted for 39.89% of the total effect in primary hospitals, while it was 20 and 19.34% in secondary and tertiary hospitals. Moderating analysis showed that respect positively influences the relationship between communication and SDM in secondary hospitals (effect = 0.327, 95% CI: 0.156–0.498), but this effect was not significant in primary (95% CI: −0.035-0.405) or tertiary hospitals (95% CI: −0.072-0.210).

**Conclusion:**

Provider-patient communication and trust were important factors influencing SDM according to healthcare providers, and respectful behaviors was key to improving communication and SDM in the secondary hospital. These suggested evidence for the development of strategies to promote SDM in the future.

## 1 Introduction

China’s healthcare system is structured around public hospitals, categorized into primary, secondary, and tertiary levels. Primary hospitals serve local communities with basic healthcare needs, and providers may have more communication with patients. While secondary hospitals handle more complex cases compared to the primary ones, and serve as the hub of healthcare system. Tertiary hospitals, often located in urban areas, provide advanced medical care, and their providers deal with higher patient volumes and more specialized treatments. Each level of hospital serves distinct roles and functions within healthcare system, and patients with different socioeconomic backgrounds can choose different hospitals according to treatment needs (1, 2).

Shared decision making (SDM) refers to a collaborative process where healthcare professionals and patients make medical decisions together based on the best available evidence, considering the patients’ values and preferences (3, 4). SDM establishes a balance between the traditional paternalistic model, where the healthcare professionals predominantly make the medical decisions, and the informed choice model, where the patients make decisions independently, while SDM forms the foundation of patient-centered healthcare (5, 6). At present, China has abandoned the traditional paternalistic medical decision-making approach and is still mainly following the informed choice model for medical decision-making (7). Increasing evidence indicated that SDM could significantly enhance health related knowledge, treatment adherence, and quality of life, and also help to reduce healthcare costs and the incidence of doctor-patient disputes, which could help reduce occupational injury risks for healthcare workers and alleviate their work-related stress (8, 9). However, despite its benefits, the implementation of SDM worldwide faces multifaceted barriers, such as healthcare policy discrepancies, institutional administration constraints, and inadequate decision-support tools, and all these problems are particularly common in developing countries (10). In China, while SDM principles have been increasingly recognized, systematic implementation remains limited due to the absence of standardized guidelines and structured decision-making procedures (11). Recent studies have identified healthcare providers’ communication competencies, attitudes toward patient autonomy, and trust-building capacities as critical yet underdeveloped components of SDM in Chinese clinical settings (12, 13). For instance, a recent Chinese study revealed that in six distinct clinical decision-making scenarios, 52.7 to 71.6% of mental health practitioners opted against SDM practices, with communication competencies and the establishment of trust-based practitioner-patient relationships emerging as the primary determining factors (14). Healthcare providers often act as the initiators of SDM, determining when and how shared decision-making begin with patients (15), but there is a lack of study on SDM process from the perspective of healthcare workers. In fact, healthcare providers’ communication skills, respectful attitudes, and levels of trust are key components of SDM practice, which urgently need study to explore their underlying mechanisms (16–18).

Patient-centered communication ensures the detailed exchange of medicine-related information (19, 20). In practice, doctors and nurses often have different types of communication with patients, in which doctors should focus more on diagnosis and treatment options, while nurses could spend more time on patient education and emotional support (21). Additionally, SDM is not solely the responsibility of doctors, but it also requires the active participation of nurses who play a vital role in adjusting treatment plans and discussing with patients to develop a consensus of physical recovery and medical care (18). Deepshikha (22) et al. in their qualitative study about racial differences in SDM, found that issues related to communication, such as providing limited emotional support and sharing limited medical information, were the most crucial factors impeding SDM. Besides, a randomized controlled trial showed that a specific communication training could effectively enhance physicians to perform SDM and reduce frustration in patients (23). Detailed information interaction helps healthcare providers better understand the patients’ needs, specify the patients’ treatment preference, and promote continuity of medical care (24). Further, doctors and nurses can explain the patients’ condition, treatment options, and their potential risks and benefits through communication (19, 25). Consequently, these could enable healthcare providers to encourage patients to participate more actively in medical decision-making. Therefore, we proposed the following research hypothesis:

*H*1: Communication between healthcare providers and patients was a direct influencing factor for SDM.

Communication between healthcare providers and patients is important to build and maintain mutual trust (26). Trust was essential for building successful patient-physician relationship, and was proven to be a significant predictor of enhancing shared information, reducing defensive practices, and improving health outcomes (27). When healthcare providers communicate effectively, it not only conveys necessary medical information but also expresses emotional empathy, which is crucial for establishing trust (28). For instance, Du et al. (29) found that doctor-patient communication could significantly influence the quality of health services and patient satisfaction, which in turn helped to build doctor-patient trust. Trust, in turn, is essential for SDM. When patients and healthcare providers build trust relationship, they are more likely to engage in open dialogs, share their preferences, and participate actively in medical decision-making. A cohort study found that patient-provider trust strengthened both behavioral changes in shared decision-making, which in turn reduced adverse medical events such as low treatment adherence (30). Based on these, we recognized that communication could improve trust and then enhance the likelihood of SDM, although the medicating effect has not been verified by empirical analysis evidence. Therefore, we proposed the following research hypothesis:

*H*2: Trust between healthcare providers and patients mediated the relationship between communication and SDM.

Respect in medical service is a critical component of patient-centered healthcare, and it often involves dignity, rights, and autonomy to patients (31). Previous studies showed that respect from healthcare providers can enhance patients’ perception of being valued and understood, thereby promoting their engagement in healthcare (32, 33). Further, existing evidence suggested that respect of medical staff could promote SDM. For instance, a study (34) found that an atmosphere of respect for the patient, will be more conducive to communication between the patient and their providers, which could improve SDM in the end. Additionally, another study (35) demonstrated that respect, such as carefully listening to patients and acknowledging their concerns, could strengthen the communication process and contribute to SDM. When healthcare providers communicate clearly, it helps patients understand their health conditions and treatment options, which is crucial for making medical decisions. Besides, respectful performance can improve the positive effects of communication by making patients feel more comfortable and willing to participate in discussions about their medical plans (36). Therefore, we proposed the following research hypothesis:

*H*3: Respect from healthcare providers moderated the relationship between communication and SDM.

**Figure 1:**
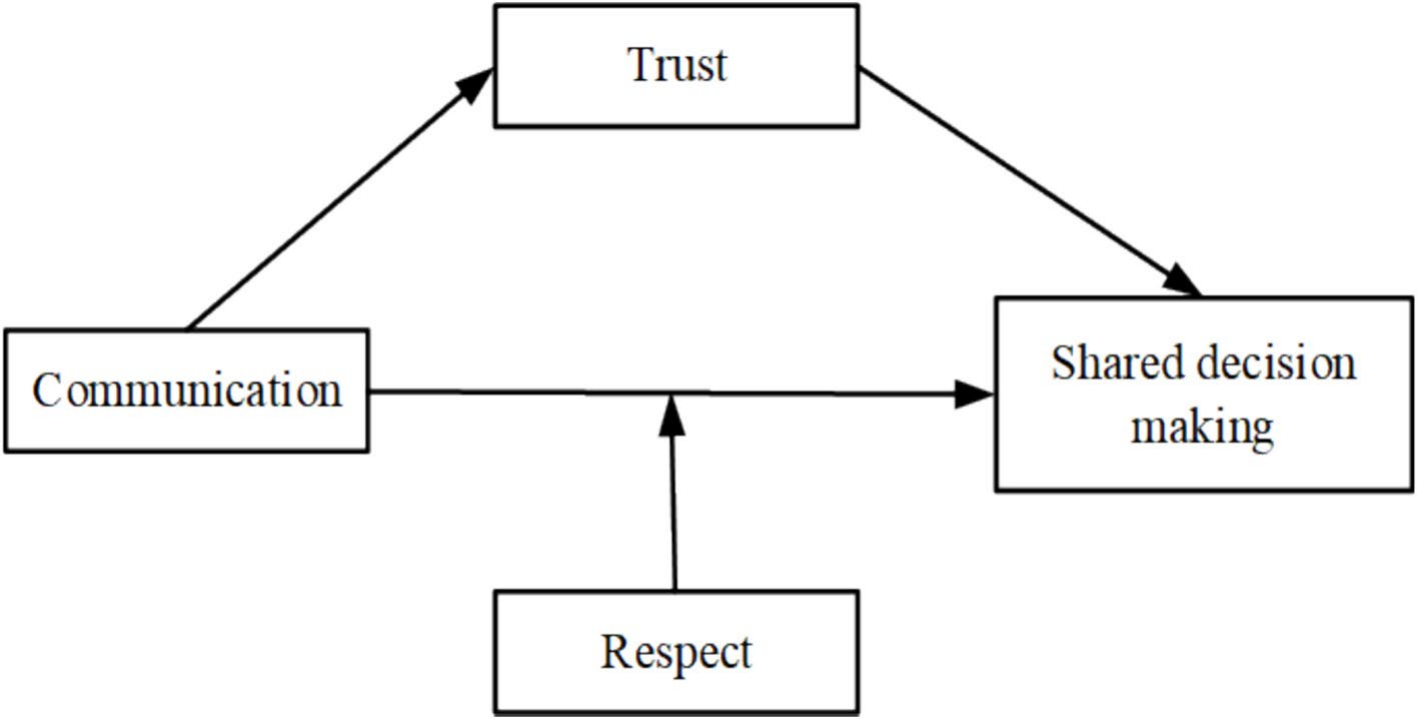
Conceptual framework of shared decision making in this study.

Based on the hypotheses above, considering the unique characteristics of primary, secondary, and tertiary hospitals, this study aimed to provide an understanding of the effects of communication, trust, and respect, on SDM (see Figure 1), which were expected to offer valuable insights for developing strategies to enhance SDM practice.

## 2 Materials and methods

### 2.1 Study design and setting

Shanghai, as one of China’s most developed and densely populated cities, exhibits significant socioeconomic and healthcare system diversity across its 16 districts, mirroring the heterogeneity found in other eastern regions (e.g., Jiangsu, Zhejiang). In April 2024, the Shanghai Municipal Health Commission issued *Guidelines on Promoting the Cultural Development of Health Services in the New Era*, explicitly emphasizing patient-centered care, enhanced doctor-patient communication, transparent medical information, and shared decision-making (SDM) in public hospitals. This policy initiative aligns with our study’s focus on healthcare providers’ communication skills, trust-building, and respectful attitudes in SDM implementation.

This cross-sectional survey was conducted from June 1, 2024, to August 30, 2024. In 2023, Shanghai had a total of 92,300 licensed physicians and approximately 113,000 registered nurses, with healthcare institutions at all levels handling 266 million patient visits throughout the year. A multistage sampling method was used to choose doctors and nurses in public hospitals in Shanghai. First, we identified 16 geographic regions based on the administrative divisions of Shanghai, and collected basic information on all public medical institutions within each region, such as number of doctors and nurses. Second, one public hospital was selected from each region, provided that the total number of doctors and nurses in each hospital exceeded 50. Then, we included 16 public hospitals, which were six primary hospitals, five secondary hospitals, and five tertiary hospitals. Next, stratified random sampling method was adopted to obtain samples, stratified according to the participants job characteristics in each hospital, and then 50 people from each hospital were selected by computer random number generation. Finally, data were collected through electronic questionnaires. The first page of the questionnaire was an informed consent form, and participants indicated their willingness to participate in the study by providing an electronic signature.

### 2.2 Participants

In the survey, we used the following criteria to include participants: (1) full-time employees of public hospitals in Shanghai, (2) working as doctors or nurses, (3) with the ability to communicate with patients, and (4) with the ability to make medical decisions. Conversely, we excluded specific participants based on the following criteria: (1) with less than 6 months of medical work experience, (2) working mainly in administrative departments with minimal involvement in medical activities, and (3) unwilling to participate in this study. A total of 800 questionnaires were distributed in target population, and 778 valid responses were received, resulting in a response rate of 97.25% in this study.

### 2.3 Measurements

The questionnaire for this study included four constructs: respect of healthcare professionals, patient-provider communication, patient-provider trust, and medical shared decision-making behavior. Besides, we collected participants’ socio-demographic information, including sex, age, marital status, and income.

**Respect of Healthcare Professionals**. We used the sub-scale from the Climate of Respect Evaluation to measure disrespectful behavior among healthcare professionals (37). This sub-scale consisted of 4 items and was initially used to assess the disrespectful behaviors performed by healthcare workers in intensive care units (37). It has been validated for use in measuring disrespect among healthcare professionals across various hospital departments. The disrespect sub-scale included four items, which measured how often that participants dismissed family concerns, talked down to patients and families, spoke disrespectfully behind their backs, and got frustrated with patients and families. All items were rated on six points measuring the frequency of these behaviors (all of the time without exception, nearly all of the time with rare exceptions, most of the time, some of the time, rarely, and never), with scores ranging from 1 to 6 after reversing code items to capture respect. In this study, the 4-item sub-scale had a Cronbach’s alpha of 0.784, indicating acceptable reliability.

**Patient-Provider Communication**. This study utilized the Physician-Patient Global Interaction Scale (PP-GIS) developed by Hodges and McIlroy (38) to evaluate the communication from the opinion of healthcare provider. This scale has been widely used to assess the level of patient-provider communication across various hospital departments (39, 40). The measurement tool included four items: responsiveness to patients’ feelings and needs (empathy), consistency in communication with patients (amount of organization), verbal expression, and non-verbal expression. Each item on the PP-GIS was measured on a five-point option: never, occasionally, sometimes, often, and always, with scores ranging from 1 to 5. In this study, the Cronbach’s alpha for the PP-GIS was 0.950, indicating excellent reliability.

**Provider-Patient Trust**. This study employed the sub-dimension “patient role” from the widely used Physician Trust in the Patient scale to measure trust (41). This sub-dimension primarily identified the healthcare professionals’ subjective attitudes toward the patients. The measurement asked healthcare professionals, “How confident are you that this patient will.,” and it included the following five items: “Provide all the medical information you need?,” “Let you know when there has been a major change in his or her condition?,” “Understand what you tell him/her?,” “Follow the treatment plan you recommend?,” and “Be actively involved in managing his/her condition/problem?.” In this study, the scale had a Cronbach’s alpha of 0.896, indicating good reliability.

**Patient-Provider Shared Decision Making**. SDM was measured using the OPTION5 Item scale, which focused on the core essential requirements when providers involved patients in decision-making (42). This scale included five dimensions: justified the work of deliberation, justified the work of deliberation as a team, informed/described options/exchange views, elicited preferences, and integrated preferences. Each item on the OPTION5 Item scale was rated on a five-point scale: never, occasionally, sometimes, often, and always, with scores ranging from 1 to 5. In this study, the Cronbach’s alpha for the OPTION5 Item was 0.946, indicating excellent reliability.

**Socio-demographic Characteristics**. This study included sex, age, job, professional ability, marriage, and income. Professional ability was determined according to the professional titles of Chinese healthcare personnel, corresponding to junior, intermediate, and high professional ability. Marriage status inquired about the respondents’ marital situation, including married and unmarried, with the latter encompassing divorced and widowed. Income was measured by the average monthly income over the past year, categorized into five levels: <5,000, 5,000–9,999, 10,000–14,999, 15,000–19,999, and >20,000 yuan.

### 2.4 Statistical analysis

For categorical variables, we used number (N) and percentage (%) to summarize the sample. For continuous variables with a nearly normal distribution, we described them using mean and standard deviation (SD). The normal distribution test was conducted by two methods: visual assessment with Q-Q plots and the Kolmogorov–Smirnov test to evaluate the probability of the data being normally distributed. After testing, the continuous variables were approximately normal distribution. These statistical analyses were performed using Stata/MP version 14.0 (StataCorp, College Station, TX, United States).

**Figure 2:**
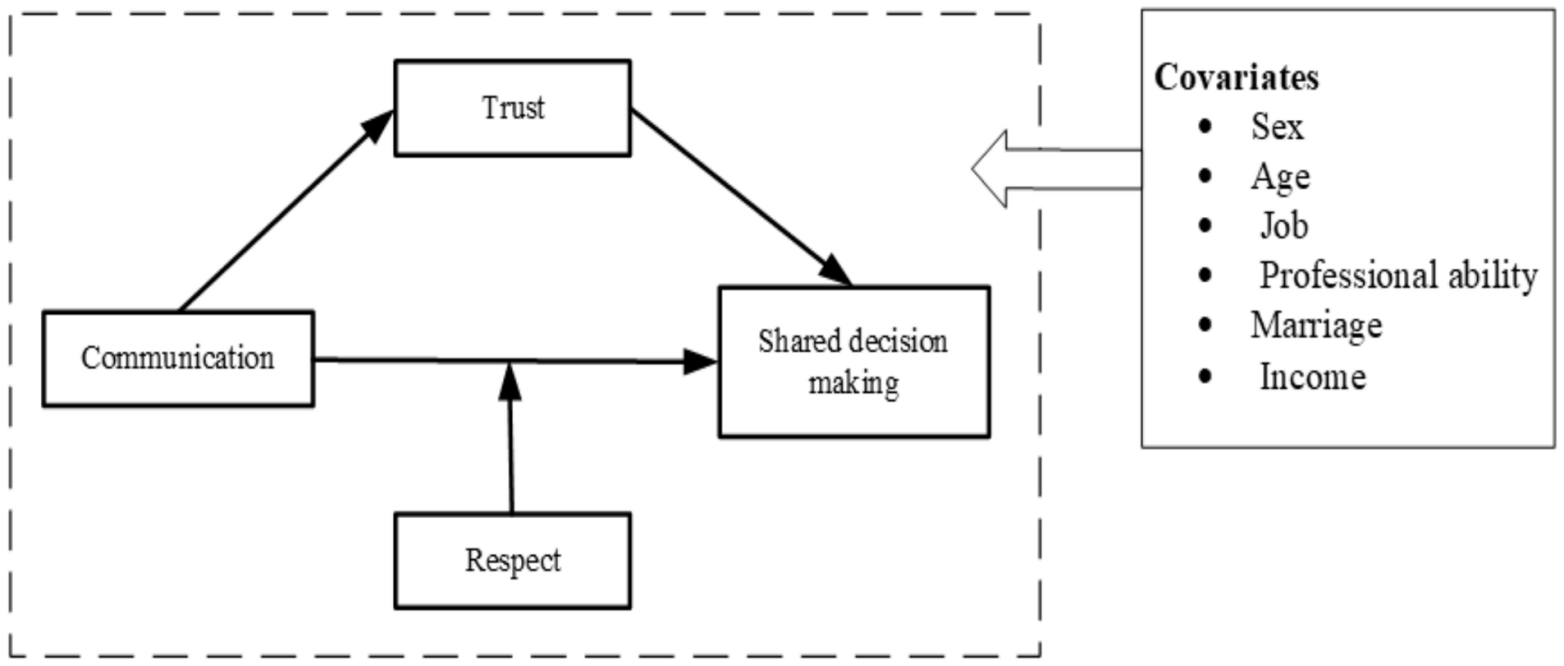
Data analysis framework based on structural equation modeling.

To account for mediating and moderating variables in the research model, we employed structural equation model (SEM) to test the hypotheses. SEM was utilized to identify latent but significant influences, providing a more comprehensive understanding of the complex mechanisms while incorporating measurement error into the research model (43). Additionally, confirmatory factor analysis (CFA) was conducted to assess the validity and reliability of the constructs, and to integrate with SEM for model improvement. We then reported factor loadings (F. L.), composite reliability (CR), average variance extracted (AVE), and discriminant validity. Model fit was assessed using the comparative fit index (CFI), Tucker-Lewis index (TLI), root mean squared error of approximation (RMSEA), and standardized root mean square residual (SRMR). Mediation and moderation models used bootstrapping (times = 2000) which was a nonparametric method that did not assume a normal distribution. Evidence of mediation and moderation was provided by a significant effect, as indicated by a 95% confidence interval (CI) that did not include zero. In SEM analysis, this study controlled the confounders of socio-demographic characteristics, to reveal the exact correlation between the study variables and SDM (see Figure 2). Also, we evaluated the importance of ‌mediating variable in explaining the relationship between independent and dependent variables, which was suggested by proportion of mediating effect (see Equation 1). The analyses were performed using Mplus version 7.4 software (Muthén & Muthén, Los Angeles, CA, United States).


(1) $$\begin{array}{l} \text{Proportion of mediating effect}=\\ \frac{\text{mediating effect}}{\text{mediating effect}+\text{direct effect}}*100\% \end{array}$$

## 3 Results

### 3.1 Characteristics of the sample

Among the 778 participants, approximately three-quarters were female (76.35%), with an average age of 39.99 years (SD = 7.50). Regarding job positions, 465 (59.77%) were doctors and 313 (40.23%) were nurses, with doctors were more than nurses in all three types of hospitals. In terms of professional ability, there were 199 (25.58%) at the junior level, 415 (53.34%) at the intermediate level, and 164 (21.08%) at the high level. Concerning marital status, 660 (84.83%) were married, while 118 (15.17%) were unmarried, divorced, or widowed. In terms of monthly income, the largest group earned 5,000–9,999 yuan (41.13%), followed by those earning 10,000–14,999 yuan (31.62%), with the smallest group earning less than 5,000 yuan, comprising only 22 (2.83%) individuals. Detailed information showed in Table 1.

**Table 1:** Demographic information of the 778 participants.

Variables	Groups	Total	Primary hospital (*N* = 262)	Secondary hospital (*N* = 270)	Tertiary hospital (*N* = 246)
Sex	Male	184 (23.65)	52 (19.85)	75 (27.78)	57 (23.17)
	Female	594 (76.35)	210 (80.15)	195 (72.22)	189 (76.83)
Age*	–	39.99 ± 7.50	41.07 ± 6.54	39.06 ± 8.67	39.85 ± 6.95
Job	Doctor	465 (59.77)	168 (64.12)	173 (64.07)	124 (50.41)
	Nurse	313 (40.23)	94 (35.88)	97 (35.93)	122 (49.59)
Professional ability	Junior	199 (25.58)	33 (12.60)	76 (28.15)	90 (36.59)
	Intermediate	415 (53.34)	183 (69.85)	130 (48.15)	102 (41.46)
	High	164 (21.08)	46 (17.56)	64 (23.70)	54 (21.95)
Marriage	Married	660 (84.83)	238 (90.84)	215 (79.63)	207 (84.15)
	Not married	118 (15.17)	24 (9.16)	55 (20.37)	39 (15.85)
Income (yuan/month)	<5,000	22 (2.83)	14 (5.34)	6 (2.22)	2 (0.81)
	5,000–9,999	320 (41.13)	151 (57.63)	120 (44.44)	49 (19.92)
	10,000–14,999	246 (31.62)	73 (27.86)	95 (35.19)	78 (31.71)
	15,000–19,999	91 (11.70)	16 (6.11)	27 (10.00)	48 (19.51)
	>20,000	99 (12.72)	8 (3.05)	22 (8.15)	69 (28.05)

*Age was described by mean±SD.

### 3.2 Reliability and validity of the constructs

All factor loadings of the items were >0.5, which showed that these items could measure the four constructs well, under the condition of the sample size being greater than 700. Additionally, all the values of the CR were >0.8, which exhibited that the constructs had good composite reliability. Besides, the values of $\sqrt{\mathrm{AVE}}s$ were more than the row and column Pearson correlation coefficient between constructs, which implied ideal discriminant validity. In brief, the study constructs exhibited good reliability and validity. See Table 2.

**Table 2:** Test of reliability and validity regarding the constructs.

Construct	F. L. range	CR	AVE	Discriminant validity			
				Respect	Comm.	Trust	SDM
Respect	0.506–0.885	0.847	0.590	**0.768**			
Comm.	0.886–0.935	0.952	0.831	0.241	**0.912**		
Trust	0.686–0.887	0.898	0.641	0.195	0.439	**0.801**	
SDM	0.789–0.940	0.948	0.784	0.196	0.538	0.506	**0.885**

The bold values were $\sqrt{\mathrm{AVE}}$ , and the lower triangular number was the Pearson correlation coefficient between constructs. Comm., communication; SDM, shared decision making; F. L., factor loading; CR, composite reliability. AVE, Average of variance extracted.

### 3.3 Test of the fitting index of the research model

The results showed that the Chi-square and degree of freedom (DF) were 287.566 and 130, respectively. It resulted in a Chi-square/DF ratio of 2.21, which was within the ideal range of 1 to 3. Additionally, the CFI and TLI values were 0.978 and 0.974, respectively, meeting the threshold standard of >0.9 (44). Moreover, the RMSEA and SRMR values were 0.039 and 0.036, respectively, both satisfying the SEM criterion of less than 0.08 (44). Therefore, these indicators suggested that the data fit the conceptual research model very well (44). See Table 3.

**Table 3:** Test of the fitting index of the research model.

Index	Criteria	Research model	Support or not
Chi-square	Small is better	287.566	Support
Degree of Freedom (DF)	Larger is better	130	Support
Chi-square/DF	3 > Chi-square/DF > 1	2.21	Support
CFI	>0.90	0.978	Support
TLI	>0.90	0.974	Support
RMSEA (90% CI)	<0.08	0.039 (0.033–0.046)	Support
SRMR	<0.08	0.036	Support

DF, degree of the freedom; CFI, comparative fit index; TLI, Tucker-Lewis index; RMSEA, root mean squared error of approximation. SRMR, standardized root mean square residual.

### 3.4 Results of the linear regression model

For primary hospitals, communication positively impacted trust between patients and doctors/nurses (b = 0.543, 95% CI: 0.389–0.669), with an R-square of 0.295 for this regression model. The occurrence of SDM was significantly and positively influenced by both doctor/nurse–patient communication (b = 0.333, 95% CI: 0.146–0.520) and trust (b = 0.407, 95% CI: 0.242–0.596), yielding an R-square of 0.423 for this regression model. In secondary hospitals, communication also had a significant positive effect on trust between patients and doctors/nurses (b = 0.302, 95% CI: 0.115–0.445). The occurrence of SDM in these hospitals was significantly positively influenced by both communication (b = 0.376, 95% CI: 0.222–0.524) and trust (b = 0.312, 95% CI: 0.184–0.455), with an R-square of 0.309 for this regression equation. Similarly, for tertiary hospitals, communication significantly positively impacted trust between patients and doctors/nurses (b = 0.401, 95% CI: 0.267–0.523). Additionally, both communication (b = 0.438, 95% CI: 0.319–0.556) and trust (b = 0.263, 95% CI: 0.113–0.403) significantly influenced SDM, with an R-square of 0.353 for this regression equation. See Table 4.

**Table 4:** Estimated results of the linear regression model.

DV	IV	Est. (b; direct effect)	S. E.	Est./S. E.	*p-*value	Bootstrap 2000 times 95% CI (bias corrected)		*R-*square
						Lower	Upper	
Primary Hospital								
Trust	Communication	0.543	0.075	7.246	<0.001	0.389	0.669	0.295
SDM	Trust	0.407	0.089	4.579	<0.001	0.242	0.596	0.423
	Communication	0.333	0.097	3.433	0.001	0.146	0.520	
Secondary Hospital								
Trust	Communication	0.302	0.082	3.664	<0.001	0.115	0.445	0.091
SDM	Trust	0.312	0.071	4.383	<0.001	0.184	0.455	0.309
	Communication	0.376	0.082	4.603	<0.001	0.222	0.524	
Tertiary Hospital								
Trust	Communication	0.401	0.066	6.064	<0.001	0.267	0.523	0.160
SDM	Trust	0.263	0.073	3.599	<0.001	0.113	0.403	0.353
	Communication	0.438	0.059	7.416	<0.001	0.319	0.556	

SDM, shared decision making; S. E., standard error; DV, dependent variable; IV, independent variable; CI, confidence interval.

### 3.5 Mediating and moderating analysis on shared decision making

In primary hospitals, trust between patients and doctors/nurses significantly mediated the relationship between communication and SDM, with a mediating effect of 0.221 (95% CI: 0.133–0.359). Moreover, the total effect of communication on SDM was the sum of the direct effect (0.333) and the indirect effect (0.221), indicating that the mediation effect due to trust constituted 39.89% of the total effect. However, respect between patients and doctors/nurses did not emerge as a significant moderator in the communication and SDM relationship (95% CI: −0.035-0.405). In secondary hospitals, the trust factor significantly mediated the link between communication and SDM, showing a mediation effect of 0.094 (95% CI: 0.054–0.157), which accounted for 20% of the total effect. Additionally, respect served as a significant moderator (95% CI: 0.156–0.498). In tertiary hospitals, trust between patients and doctors/nurses also played a significant mediating role in the communication-SDM relationship, with a mediation effect of 0.105 (95% CI: 0.052–0.179), representing 19.34% of the total effect. Nonetheless, respect did not act as a significant moderator in the relationship between communication and SDM (95% CI: −0.072-0.210). See Table 5.

**Table 5:** Results of mediating and moderating analysis.

Effects	Est. size	S. E.	Est./S. E.	*p-*value	Bootstrap 2000 times 95% CI (bias corrected)	
					Lower	Upper
Primary Hospital						
Communication→Trust→SDM	0.221	0.056	3.974	<0.001	0.133	0.359
Communication×respect→SDM	0.185	0.113	1.637	0.102	−0.035	0.405
Secondary Hospital						
Communication→Trust→SDM	0.094	0.026	3.685	<0.001	0.054	0.157
Communication×respect→SDM	0.327	0.085	3.831	<0.001	0.156	0.498
Tertiary Hospital						
Communication→Trust→SDM	0.105	0.031	3.378	0.001	0.052	0.179
Communication×respect→SDM	0.069	0.072	0.958	0.338	−0.072	0.210

→ meant the mediating effect, and × meant moderating effect. SDM, shared decision making; S. E., standard error; CI, confidence interval.

## 4 Discussion

SDM is crucial for addressing the information asymmetry inherent in healthcare services, as it can ensure both providers and patients fully understand the disease condition, treatment plan, and the expected outcomes. However, few studies have explored the mechanism of SDM from the perspective of healthcare provider who plays a leading role in the SDM process, so it has not yet understood how to promote SDM in the process of medical services. In order to fill this research gap, this study explored the effects of communication, trust, and respect on SDM at different levels of public hospitals, which could provide evidence for the development of strategies to promote SDM in the future.

First of all, this study developed a conceptual framework for SDM by literature review, which proposed the impact pathways of SDM in theory. Furthermore, we selected developed measurement tools and conducted confirmatory factor analysis, which revealed that the constructs in our study had good reliability and validity, indicating the suitability of these measurement tools for this study. Additionally, we employed SEM to examine the theoretical framework, and found that all model fit indexes met statistical requirements. These findings have significant implications, providing a basic framework for future SDM studies and practice.

The regression analysis indicated that for healthcare providers in China’s three-tier public hospital system, communication and trust were crucial factors influencing SDM from the perspective of healthcare providers. This finding was in line with Derrington et al. (45) that found SDM depended on high-quality communication between the physician and patients. The detailed exchange of information in communication process ensures that patients and medical staff can be well-informed about disease condition, the available treatment options, and the associated risks and benefits, which empowers them to participate in the decision-making process (46). Besides, trust between healthcare providers and patients was found to be another significant factor influencing SDM. Trust reduces psychological barriers and fosters a collaborative environment where physicians feel comfortable to provide the comprehensive and feasible disease treatment and health promotion plans (47, 48). Furthermore, the coefficient of determination from the regression analysis revealed that trust and communication between healthcare providers and patients accounted for over 30% of the variance in SDM across the three types of public hospitals. This finding indicated that enhancing trust and communication could potentially increase the likelihood of healthcare providers engaging in SDM by more than 30%. However, the occurrence of communication and trust can be complicated due to deeply ingrained cultural values in China. On the patient side, persistent paternalistic health beliefs may lead to reluctance in challenging physicians’ opinions, whereas younger generations increasingly demand autonomy—creating trust-building challenges (49). Conversely, healthcare providers trained in expert-authority model, which may perceive SDM as time-consuming rather than beneficial (50). Therefore, health-related institutions should invest to conduct mandatory communication skill training for all physicians, focusing on information structuring and emotional resonance. In addition, the public hospitals should build standardized trust metrics in daily management, and carry out regular monitoring and assessment of trust levels, by which SDM can be effectively realized in real world.

The mediation model analysis indicated that, in all public hospitals, effective communication positively impacted trust between patients and healthcare providers, which in turn promoted the occurrence of SDM. This finding highlighted that communication could directly influence trust, and it subsequently enhanced SDM practice for the healthcare providers. Effective communication is not only the ability of healthcare professionals to convey information about diagnoses, treatment options, and potential risks and benefits, but also includes emotional interactions and compassionate expression (51). These are vital for patients to feel concerned, understood, and warm, which then contributes to form mutual trust and encourage SDM in medical service process (52). In further analysis, this study revealed that the proportion of the mediation effect to the total effect varies across different levels of public hospital. In primary, secondary, and tertiary institutions, the mediation effect accounted for 39.89, 20, and 19.34% of the total effect, respectively. In secondary and tertiary hospitals, while the mediation effect was still significant, it was relatively lower compared to primary hospitals. In the primary healthcare, under China’s policy mandating ‘primary care first’, patients with chronic conditions establish long-term relationships with primary providers (53). This relational continuity enables cumulative communication to foster deep affective trust, so trust mediates nearly 40% of communication’s effect on SDM, as mutual understanding transforms dialog into SDM (29). Secondary hospitals often face severe patient-provider ratio imbalances, creating transactional rather than relational encounters, and communication becomes task-oriented, which then could inhibit trust development (54). In secondary hospital, although trust remained a significant mediator, its reduced role (20%) reflects more complex and specialized nature of care in these settings. In tertiary hospitals, complex cases involving multiple specialists can decline individual communication effects, and more utilities of decision aids may reduce reliance on interpersonal trust, both of which could weaken the mediating effect (9, 11). Therefore, though improving communication remains momentous, additional strategies to build trust should also be essential to enhance SDM in three-level hospitals. Primary hospitals should implement relationship continuity incentives and community-based trust circles, while secondary and tertiary hospitals should develop time-sensitive communication toolkits, and combine expertise-transparency displays with decision aid integration, which could be beneficial to achieve SDM practice.

The analysis of the moderation model revealed that, for healthcare providers in secondary public hospitals, respect positively moderated the relationship between patient-provider communication and SDM. Secondary hospitals typically cater to diverse patient population with different health conditions, which requires a balance between specialized care and general medical services (55). In such settings, respect from healthcare providers can enhance communication by creating a more open and easy-going dialog environment, which in turn facilitates more effective SDM (56). Besides, respect counteracts traditional hierarchical form which medical staff have the monopoly authority in healthcare process, and it could reduce power asymmetry, enabling equitable dialog essential for SDM (57). However, this moderating effect was not significant among healthcare providers in primary and tertiary hospitals. In primary hospital, patients often have more frequent and ongoing interactions with their providers, in which patients will naturally feel get much respect regardless medical staff have not great respect attitude, so it potentially diminishes the observable moderating effect of respect (58). In tertiary hospital, healthcare often involves complex medical conditions and advanced treatments, where clinical expertise and the clarity of medical information could take precedence over interpersonal communication (59). Additionally, the higher patient turnover and the more transactional nature of interactions in tertiary settings can limit the extent to which respect moderate the relationship of communication and SDM (60). Therefore, public hospitals, especially the secondary, should develop policies that promote a culture of respect among healthcare providers, which includes patient-centered service and communication training, and regular feedback mechanisms to assess and improve provider-patient communication.

However, some limitations should be mentioned in this study. Firstly, consistent with all cross-sectional designs, our SEM analysis identified statistically significant associations but cannot prove causation. While we employed anchored scale items and controlled for key confounders, the directionality of relationships between communication competence, trust, and SDM implementation remains theoretically inferred rather than empirically demonstrated. While the findings suggested significant associations, longitudinal studies were needed to confirm the causal pathways and to observe changes over time. Secondly, the study was conducted in public hospitals in Shanghai, China, which may limit the generalizability of the findings to other regions or healthcare systems. This study, based on the public hospitals in Shanghai, explored the influencing factors of SDM practice in developed regions. However, different cultural, economic development tiers, and medical systems across diverse regions could influence the relationship of communication, trust, and SDM. Therefore, the future studies should include more regions to examine these results. Thirdly, this study predominantly centered on the impact of communication, trust, and respect on SDM but failed to comprehensively account for other salient factors that may influence SDM, such as healthcare provider workload, time constraints, and specific clinical contexts. To gain a more profound comprehension of the SDM influencing mechanism, future study should include a broader range of variables in research framework.

## 5 Conclusion

From the perspective of doctors and nurses, this study established a feasible conceptual framework for SDM in public hospital of China. Subsequently, SEM was employed to validate the following hypotheses: (1) communication between providers and patients was a direct influencing factor for SDM; (2) trust between providers and patients mediated the effect of communication on SDM; (3) respect from providers moderated the relationship between communication and SDM, although this finding was not applicable to primary and tertiary hospitals. These findings provided valuable evidence for developing strategies to improve SDM practice within public healthcare system.

## Data Availability

The raw data supporting the conclusions of this article will be made available by the authors, without undue reservation.
